# One pot synthesis of poly m-toluidine incorporated silver and silver oxide nanocomposite as a promising electrode for supercapacitor devices

**DOI:** 10.1038/s41598-024-84848-5

**Published:** 2025-01-21

**Authors:** Doaa Essam, Ashour M. Ahmed, Ahmed A. Abdel-Khaliek, Mohamed Shaban, Mohamed Rabia

**Affiliations:** 1 Nanomaterials Science Research Laboratory, Chemistry Department, Faculty of Science, Beni-Suef University, Beni-Suef, Egypt; 2 Physical Chemistry Laboratory, Chemistry Department, Faculty of Science, Beni-Suef University, Beni-Suef, 62514 Egypt; 3 Physics Department, College of Science, Imam Mohammad Ibn Saud Islamic University (IMSIU), 11623 Riyadh, Saudi Arabia; 4 Nanophotonics and Applications Lab, Physics Department, Faculty of Science, Beni-Suef University, Beni-Suef, 62514 Egypt; 5 Physics Department, Faculty of Science, Islamic University of Madinah, P. O. Box: 170, 42351 Al Madinah Al Monawara, Saudi Arabia

**Keywords:** Poly (m-tolidine), Ag-Ag_2_O, Specific capacitance, Pseudocapacitor, Specific energy density, Energy science and technology, Materials science, Nanoscience and technology

## Abstract

The design and fabrication of novel electrodes with strong electrochemical responses are crucial in advanced supercapacitor technology. In this study, a poly(m-toluidine)/silver-silver oxide (PMT/Ag-Ag_2_O) nanocomposite was prepared using the photopolymerization method. Various characterization techniques were employed to analyze the prepared nanomaterials. The resulting structure of Ag-Ag_2_O minimizes ion diffusion distances, increases active sites, and accelerates redox reactions. The electrochemical response of PMT and PMT/Ag-Ag_2_O electrodes was evaluated in three different electrolyte solutions (Na_2_SO_4_, H_2_SO_4_, and HCl). The specific capacitance of PMT/Ag-Ag_2_O nanocomposite was found to be higher than that of PMT alone. Among the tested electrolytes, HCl exhibited the highest specific capacitance of 443 F g^−1^ at a gravimetric current density of 0.4 A g^−1^, surpassing H_2_SO_4_ (104 F g^−1^) and Na_2_SO_4_ (32 F g^−1^). Also, the PMT/Ag-Ag_2_O nanocomposite has demonstrated good cycling stability. It exhibited a high specific power density of 156 W Kg^−1^ and a specific energy density of 1.8 Wh Kg^−1^. These results highlight the potential of the prepared PMT/Ag-Ag_2_O nanocomposite as a nanoelectrode material for high-performance supercapacitors.

**Supplementary Information:**

The online version contains supplementary material available at 10.1038/s41598-024-84848-5.

## Introduction

Supercapacitors are a type of energy storage device that was developed and manufactured to solve the problem of energy storage systems^1,2^. This is because of their large power density and excellent cycle life^3,4^. Based on the store charge, supercapacitor energy storage devices can be put into one of three groups: electric double-layer capacitors (EDLCs), pseudocapacitors, and hybrid electrodes^5–7^. The mechanisms of storing energy in EDLCs depend on the physical storage charges (physisorption) formed by forming an electric double layer (EDL) at the interface between the electrolyte and electrode material^8–10^. The formation of a double layer occurs when negatively charged electrolytes adsorb on positively charged electrodes and vice versa. Carbonaceous materials are highly preferred as electrodes for EDLCs due to their significantly high electrical conductivity, porosity, and surface area^6,8,11^. Their higher power density is a result of the electrolyte’s fast ions’ diffusion to the electrode surface. However, EDLCs have relatively low specific energy in comparison to batteries^12^. In pseudocapacitive capacitors, the charge is stored through redox Faradaic reactions, which have been investigated to boost energy density^13^. Pseudocapacitive materials possess the capability to have a larger C_sp_ and energy density (up to 10 Wh kg^-1^) than that of EDLCs^14^. Conducting polymers (CPs), metals, and transition metal oxides are examples of pseudocapacitors^15^. One of the numerous CPs is polyaniline, which has undergone the most investigation^16^. The main obstacle to the practical application of polymers is their insufficient cycling stability, leading to low capacity retention. This issue arises from the expansion and shrinkage of the polymer caused by the oxidation and reduction of ions within the polymer’s backbone^17^.

Recently, transition metal oxides (TMOs) used in electrode structures have boosted reversible redox processes, energy density, and capacitance^18^. Vanadium oxide (V_2_O_5_), manganese oxide (MnO_2_), and iron oxide (Fe_2_O_3_) are different transition metal oxide-based electrodes^19–21^. Even if the C_sp_ is increased by using TMOs alone within the electrode framework, their limited potential window allows them to considerably reduce energy density^15^. These supercapacitors’ specific energy is still considerably less than that of batteries^22^. According to previous studies, Ag_2_O nanoparticles are extremely conductive and thus appropriate for utility in energy and electrochemical applications^23,24^. The features of silver oxide, including its porous shape, high conductivity, acceptable thermal stability, and good wettability, make it an interesting electroactive material for supercapacitor applications^23,25^. Silver oxide’s ability to change and have several oxidation numbers (+ 1, + 2). It has several forms, including AgO, Ag_2_O_2_, Ag_2_O_3_, Ag_3_O_4_, Ag_4_O_3_, and Ag_2_O^23,24^. Among them, Ag_2_O has been the most stable phase.

On the other hand, Poly m-toluidine (PMT) has good conductivity, electrochromic properties, thermal stability, and environmental stability^26,27^. PMT polymer has approximately identical electrochromic and redox properties to polyaniline (PANI), and it has limited basicity with limited solubility in water. Additionally, it is inexpensive, ecologically beneficial, simple to make, very chemically stable, and repeatable^28^. Furthermore, it can polymerize to produce well-known CPs with semiconducting characteristics and strong optical absorbance^29^. Hence, it has been used in several applications, including supercapacitors, hydrogen generation, photon sensing, and an optoelectronic photodetector^4,29–32^.

Compared to the PANI polymer discussed in previous work^33^, the PMT polymer material used in this study is innovative. PMT features methyl groups on the phenyl rings, which contribute additional electrons. The incorporation of these functional groups into the aromatic ring increases the surface area of the electroactive sites, leading to faster charging / discharging as well as improved polymer stability^34,35^. Sivakumar et al. observed that poly ortho-toluidine (POT), which has an electron-donating methyl group on its phenyl ring, exhibits increased capacitance and lower resistance compared to unsubstituted PANI^36^. The polymer forms more quickly when multiple oxidants are present in the reaction compared to using a single oxidant. Additionally, adding a second oxidant to the polymerization process can significantly enhance the properties of the polymer compared to using just one oxidant^37^. Fanxin et al. observed increased specific capacitance and cycle stability when APS was used in conjunction with K_2_Cr_2_O_7_ and FeCl_3_ as mixed oxidants for PANI, compared to using a single oxidant^38^. Therefore, using Ag_2_O with APS as two oxidants in this study facilitates a quicker polymer synthesis procedure with improved capacitance. The content of silver in the final nanocomposite increases when APS and silver nitrate are used together, as the polymerization process benefits from the combined effect of both oxidants. Additionally, the electrical conductivity is improved by this oxidation mixture, with the enhancement in conductivity linked to better electron transport^39^. Aya et al. used the same polymer with Co–Ni as the active material to enhance the capacitance for supercapacitors^29^. Their nanocomposite structure includes PMT polymer, cobalt nickel oxide, and cobalt carbonate hydroxide hydrate. However, this approach has several drawbacks. The preparation method involves hydrothermal treatment for 9 h at 120 °C, followed by calcination for 3 h at 400 °C, polymerization for 2 h, and drying for 12 h in an oven at 80 °C. These multi-step processes are complex, expensive, and time-consuming. Additionally, issues such as agglomeration, production difficulties, and challenges with electrical conductivity arise from these steps. The resulting provides low specific capacitance and limited specific energy.

In supercapacitors, the electrolyte is a vital and significant component that is crucial for the balancing of charge transfer between the two electrodes^6^. The electrolyte must be chosen carefully for supercapacitor devices to be secure and perform well. Aqueous electrolytes have been widely used in research and development compared to organic electrolytes and ionic liquids^40^. In comparison to organic and ionic electrolytes, aqueous electrolytes have better conductivities, which advantage to supercapacitors by reducing equivalent series resistance and enhancing power delivery^6,41^. Also, aqueous electrolytes are simpler to handle in the lab than organic electrolytes and ionic liquids, which need purification processes.

This work introduces several novel elements to enhance the significance of supercapacitor applications. The study breaks conventional practices by utilizing poly m-toluidine (PMT) instead of polyaniline (PANI) in supercapacitor applications. The novel use of PMT/Ag-Ag_2_O nanocomposites as electroactive materials is explored for the first time. PMT offers improved conductivity, thermal stability, and easy synthesis. The incorporation of an Ag/Ag_2_O structure enhances surface area, charge transport efficiency, and reaction sites. The fabrication process using the photo-polymerization method is simple and reproducible. A novel approach to controlling high yield and energy-storing polymer nanostructures is provided by mixed oxidants. In comparison to utilizing only one oxidant, introducing a second oxidant during the polymerization process can greatly improve the polymer’s characteristics. Characterization techniques such as XRD, TEM, XPS, SEM, and FTIR were employed. Electrochemical tests in different electrolytes, including Na_2_SO_4_, H_2_SO_4_, and HCl. The result demonstrated the high performance and stability of PMT/Ag-Ag_2_O nanocomposites. Notably, the HCl electrolyte exhibited higher capacitance (443 F g^-1^ at 0.4 A g^-1^). Furthermore, the nanocomposite material exhibited good stability. These findings position the PMT/Ag-Ag_2_O nanocomposite as a promising candidate for supercapacitor applications.

## Experimental section

### Materials and preparation methods

Silver nitrate (AgNO_3_) and m-toluidine (C_6_H_4_CH_3_NH_2_) were obtained from the Rankem Company, India. Ammonium persulfate (APS) ((NH_4_)_2_S_2_O_8_) was acquired from Winlab Company, USA. Graphite powder was acquired from Piochem Company, Egypt. Nafion solution (5 wt. % in methanol) was obtained from Sigma Aldrich, USA. Acetic acid (CH_3_COOH), hydrochloric acid (HCl), sodium sulfate (Na_2_SO_4_), and sulfuric acid (H_2_SO_4_) are bought from the El-Naser Company in Egypt.

Poly(m-toluidine) (PMT) was synthesized using the photo-polymerization method. Initially, 0.12 M of m-toluidine was dissolved in a solution containing 0.5 M CH_3_COOH. Simultaneously, 0.12 M of APS (an oxidizing agent) was dissolved in a separate solution of 0.5 M CH_3_COOH. Then, APS solution was added to the m-toluidine solution under UV irradiation and allowed to react for 2 h. To ensure a complete reaction, the mixture was left undisturbed for an additional 12 h. Subsequently, the resulting mixture was filtered to separate the PMT powder. The obtained PMT was washed several times and then dried at 60 °C for 24 h, resulting in the final product of pristine PMT powder.

The synthesis of the PMT/Ag_2_O@Ag nanocomposite followed a similar procedure to the pristine PMT preparation. However, there were some modifications to the process. In this case, the oxidation solution contained 0.06 M AgNO_3_. Additionally, a lower concentration of APS (0.06 M) was used compared to the pristine PMT synthesis (0.12 M). The process began by dissolving 0.12 M m-toluidine in 0.5 M CH_3_COOH. Simultaneously, a solution containing 0.06 M AgNO_3_, 0.06 M APS, and 0.5 M CH_3_COOH was prepared. These solutions were mixed under UV irradiation for 2 h and then left undisturbed for 24 h. The mixture was filtered to separate the nanocomposite powder, which was subsequently washed thoroughly and dried to obtain PMT/Ag_2_O-Ag nanocomposite. A simplified illustration of the synthesis process is presented in Figure S1.

The photopolymerization mechanism involves AgNO_3_ and ammonium persulfate (NH_4_)_2_S_2_O_8_ (APS). While AgNO_3_ acts as a photo-initiator, APS does not. AgNO_3_ reacts with APS to form Ag/Ag_2_O. The reactions are represented by Eqs. (1) and (2). Ag_2_O, with its negative charge and photocatalytic properties due to its semiconductivity and small bandgap, facilitates the activation of additional AgNO_3_^42,43^. This activation, in turn, initiates the oxidation of the m-toluidine monomer to form PMT under light. Previous literature details AgNO_3_'s ability to drive photopolymerization reactions^33^.

1 $${{(\text{NH}}_{4})}_{2}{\text{S}}_{2}{\text{O}}_{8}+2 {\text{AgNO}}_{3} \to {\text{Ag}}_{2}\text{O}+({\text{NH}}_{4}{)}_{2}{\text{SO}}_{4}+2\text{ N}{\text{O}}_{2}+{\text{SO}}_{4}^{2-}$$

2 $${\text{Ag}}_{2}\text{O}+\text{ O }\to \text{Ag}+ {\text{O}}_{2}$$

### Characterization of synthesized materials

Several techniques were employed to gain valuable insights into the structural, compositional, chemical, and morphological characteristics of the prepared nanomaterials. X-ray diffraction (XRD) and Fourier-transform infrared spectroscopy (FTIR) were utilized to examine the structural and compositional properties of the pristine PMT and PMT/Ag-Ag_2_O nanocomposite. XRD analysis was conducted using a PANalytical X-ray diffractometer. FTIR spectroscopy was performed using a Bruker-Vertex 70 instrument. X-ray photoelectron spectroscopy (XPS) was employed to determine the chemical state of the materials using an Axis Ultra DLD instrument. Scanning electron microscopy (SEM) and transmission electron microscopy (TEM) were utilized for morphological analysis, utilizing an Axioskop 40 POL microscope and a JOEL JEM-2010 instrument, respectively.

### Fabrication of two-electrode system supercapacitor

For all electrochemical tests conducted, a symmetric two-electrode configuration was employed. This means that the same type of electrode material was used for both terminals of the supercapacitor. To prepare the catalyst ink, a mixture of 20 mg of ground active material (PMT or PMT/Ag-Ag_2_O), 0.025 mg of graphite powder as an additive, 50 µL of Nafion, and 300 µL of ethanol was stirred for 24 h to obtain a cohesive catalyst ink. In general, the addition of graphite alongside active materials in supercapacitors serves various significant roles, including providing structural support, enhancing surface area, improving cycling stability, and preventing agglomeration. Approximately 56 µL of the homogeneous slurry was then cast onto a gold sheet with an area of 1 × 1 cm^2^. The Au sheet acted as a current collector. The resulting nanocomposite material electrode on the Au sheet had an approximate thickness of 4.73 μm. In addition, three pieces of filter paper were soaked in the corresponding electrolytes overnight, including 0.5 M Na_2_SO_4_, 0.5 M H_2_SO_4_, or 0.5 M HCl. Subsequently, one piece of filter paper was inserted between the two fabricated electrodes, serving as a separator.

### Electrochemical studies

All electrochemical measurements of the PMT and PMT/Ag-Ag_2_O nanocomposite were operated out in two symmetric-electrode systems using an electrochemical workstation (CHI 660E; CH Instruments, China). Galvanostatic charge/discharge (GCD) calculations at various current densities, cyclic voltammetry (CV) at various scan rates, and a voltage window applied between 0 and 1 V in various aqueous electrolyte solutions were performed. Electrochemical impedance spectra (EIS) were obtained using a 10 mHz to 100 kHz frequency range. Figure S2 illustrates the schematic representation of the electrochemical measurement setup.

## Results and discussions

### Structural and morphological studies

**Fig. 1:**
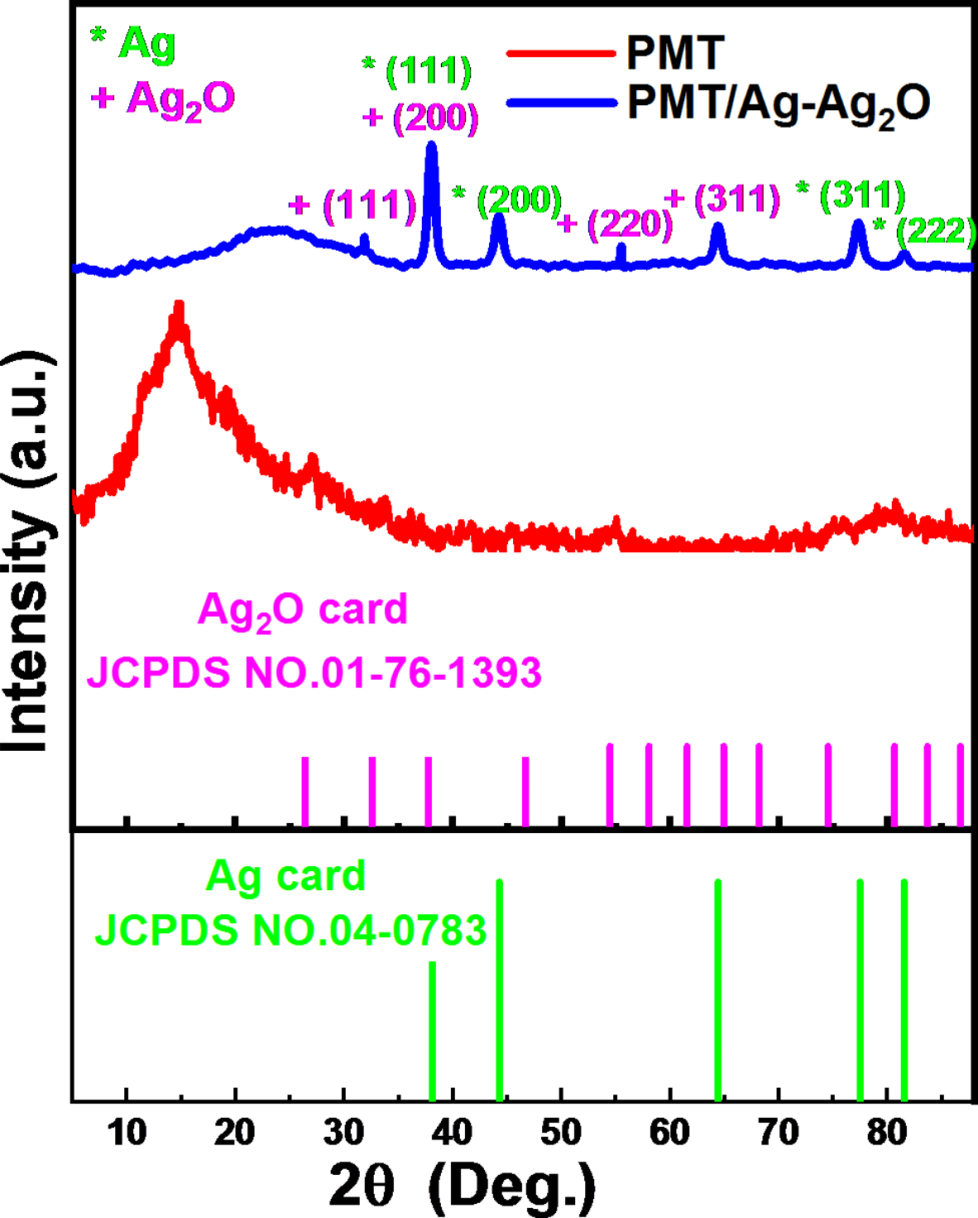
XRD of pristine PMT and PMT/Ag-Ag2O nanocomposite with standard XRD patterns of Ag and Ag_2_O cards.

The XRD technique is utilized to study the prepared nanocomposite’s crystal structure. Figure 1 provides the X-ray diffraction bands of the pristine PMT and PMT/Ag-Ag_2_O nanocomposite. Also, the standard XRD patterns for Ag and Ag_2_O are presented in Fig. 1. For the PMT, the broad diffraction pattern observed in the selection range of 2θ = 10°—20° indicates the pristine PMT exhibits an amorphous structure^44^. The two diffraction peaks are observed at 2θ = 14° and 25°, attributed to the perpendicular and parallel periodicities of the polymer chain, respectively^44,45^.

For the PMT/Ag@Ag_2_O, it is evident that the characteristic peak of the PMT is clear at 2θ = 23°. The peaks pointed at 2θ = 32.89°, 38.1°, 55.2°, and 64.8°, corresponding to (111), (200), (220), and (311), respectively, are attributed to the Ag_2_O cubic structure according to [JCPDS card No. 01- 76–1393]^46^. Also, the peaks pointed at 2θ = 44.5°, 77.34°, and 81.3° corresponded to the Ag metallic phase (200), (311), and (400) planes, respectively, with the face-centered cubic structure [JCPDS card No. 04–0783]^47^.

The diffraction peaks of both silver (Ag) and silver oxide (Ag_2_O) overlap significantly at 2θ = 38.14°. This overlapping behavior confirms the existence of an Ag-Ag_2_O hybrid structure in the nanocomposite material^48^. The diffraction peak observed at 2θ = 38° could be ascribed to the metallic structure of Ag and/or Ag_2_O, confirming the presence of Ag in the nanocomposite. These results align with the information provided in JCPDS cards No. 65–2871 and 04–0783 for Ag, and No. 41–1104 for Ag_2_O^49,50^.

The crystallite size (D) for PMT/Ag-Ag_2_O nanocomposite was calculated by the Debye–Scherrer equation, given by eqution 3.

3 $$\text{D}=\text{K} \uplambda/ \upbeta \cos\uptheta$$

where K is constant depending on the crystalline shape and reported to be 0.94, λ is the X-ray wavelength, CuKα radiation (1.5406 Å), β which denotes the full width of the half maximum, and finally θ refers to the incident angle. Depending on eqution 3, the nanocomposite crystallite size is calculated to e 11.82 nm at the highly intense peak (2θ = 38.14°).

**Fig. 2:**
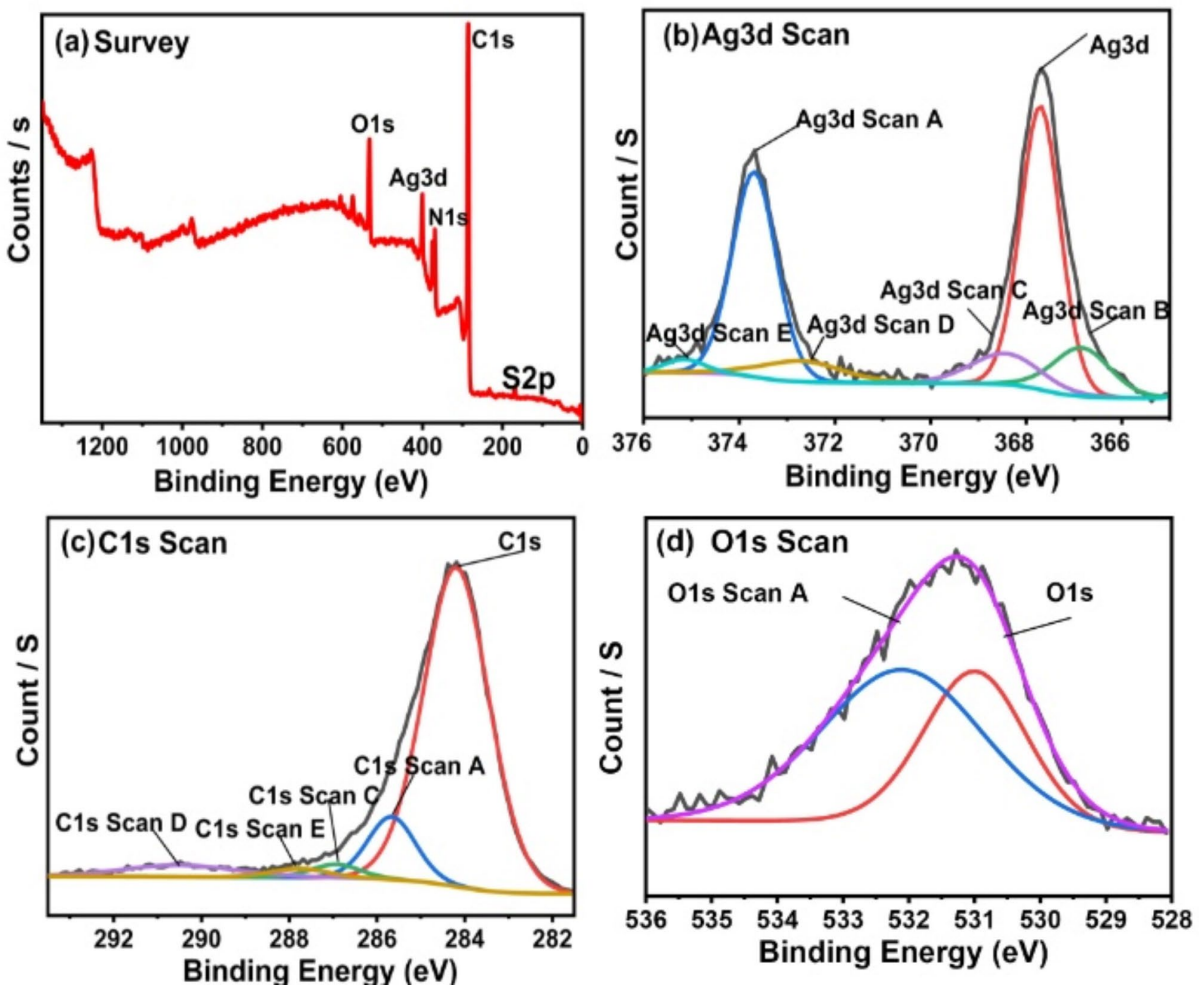
XPS characterization of the PMT/Ag-Ag_2_O nanocomposite (**a**) full survey, (**b**) Ag3d (**c**) O1s, and (**d**) C1s spectra.

The composition of the elements and their bonding state in PMT/Ag-Ag_2_O nanocomposite were demonstrated by XPS spectra, as shown in Fig. 2. The full survey data results of the nanocomposite are given in Fig. 2a, which displays a C1s major peak at a binding energy of 285.81 eV with a 76.98% atomic ratio. Furthermore, the peak of O1s is observed at 532.54 eV, providing an atomic ratio of about 8.76%, but the N1s peak is indicated at 400.26 eV, with an atomic ratio of 11.59%. Eventually, the Ag3d peak was observed at the binding energy of 368.96 eV and possessed an atomic ratio of 1.333%. As shown in Fig. 2b, the Ag 3d high-resolution XPS spectrum primarily exhibits two strong bands at 367.71 eV and 373.61 eV. Each of these bands can also be deconvoluted into a pair of subpeaks. The band at 367.71 eV (with an atomic ratio of 40.56%) is split into subpeaks at 368.43 eV and 366.8 eV, corresponding to Ag 3d_5/2_ for Ag nanoparticles (Ag NP) and Ag⁺. The band at 373.61 eV (with an atomic ratio of 32.72%) is divided into subpeaks at 375.15 eV and 372.75 eV, corresponding to Ag 3d_3/2_ for Ag NP and Ag⁺^51^. The peaks at 368.43 eV and 375.15 eV are attributed to Ag⁰ (3d_5/2_ and 3d_3/2_) with atomic ratios of 7.75% and 1.88%, respectively^51^. The other peaks at 366.8 eV and 372.75 eV are assigned to Ag⁺ (3d_5/2_ and 3d_3/2_) with atomic ratios of 9.91% and 7.18%, respectively. The findings identified two distinct species of silver (Ag_2_O and Ag), along with the reports published^52,53^.

Shaimaa et al. investigated the XPS of Ag@Ag_2_O nanostructures^53^. They found that the Ag3d XPS peaks for Ag⁰ (3d_5/2_ and 3d_3/2_) are located at 367.98 eV and 373.96 eV, respectively. In contrast, the peaks for Ag^+^ (3d_5/2_ and 3d_3/2_) are found at 367.38 eV and 373.6 eV, respectively. Aline et al. analyzed the XPS of Ag/AgO films prepared by spray-pyrolysis. They identified binding energies of 367.42 and 373.5 eV corresponding to Ag^2+^, while those at 367.95 eV and 373.97 eV were attributed to metallic silver^54^. Also, Mishra et al. noted the presence of two silver in the form of Ag (368.3 eV) and Ag_2_O (367.6 eV) based on the deconvolution of Ag 3d XPS^55^. Additionally, there are several studies that are similar to our experimental and fitted XPS analysis data of the Ag spectrum^53,56–61^

The C1s spectrum is shown in Fig. 2c. It is composed of four different carbon species: C–C/C = C (286.5 eV), C-S (287.3 eV), C-N (287.9 eV), and C-O (288.5 eV)^52^. The peak of O1s in the O1s spectrum, Fig. 2d, is divided into two sub-peaks, one at 530.99 eV with an atomic ratio of 39.15% and the second at 532.08 eV showing a 60.85% atomic ratio. Eventually, the findings show two distinct silver species, Ag_2_O and Ag, which agree with much of the published literature^47^.

**Fig. 3:**
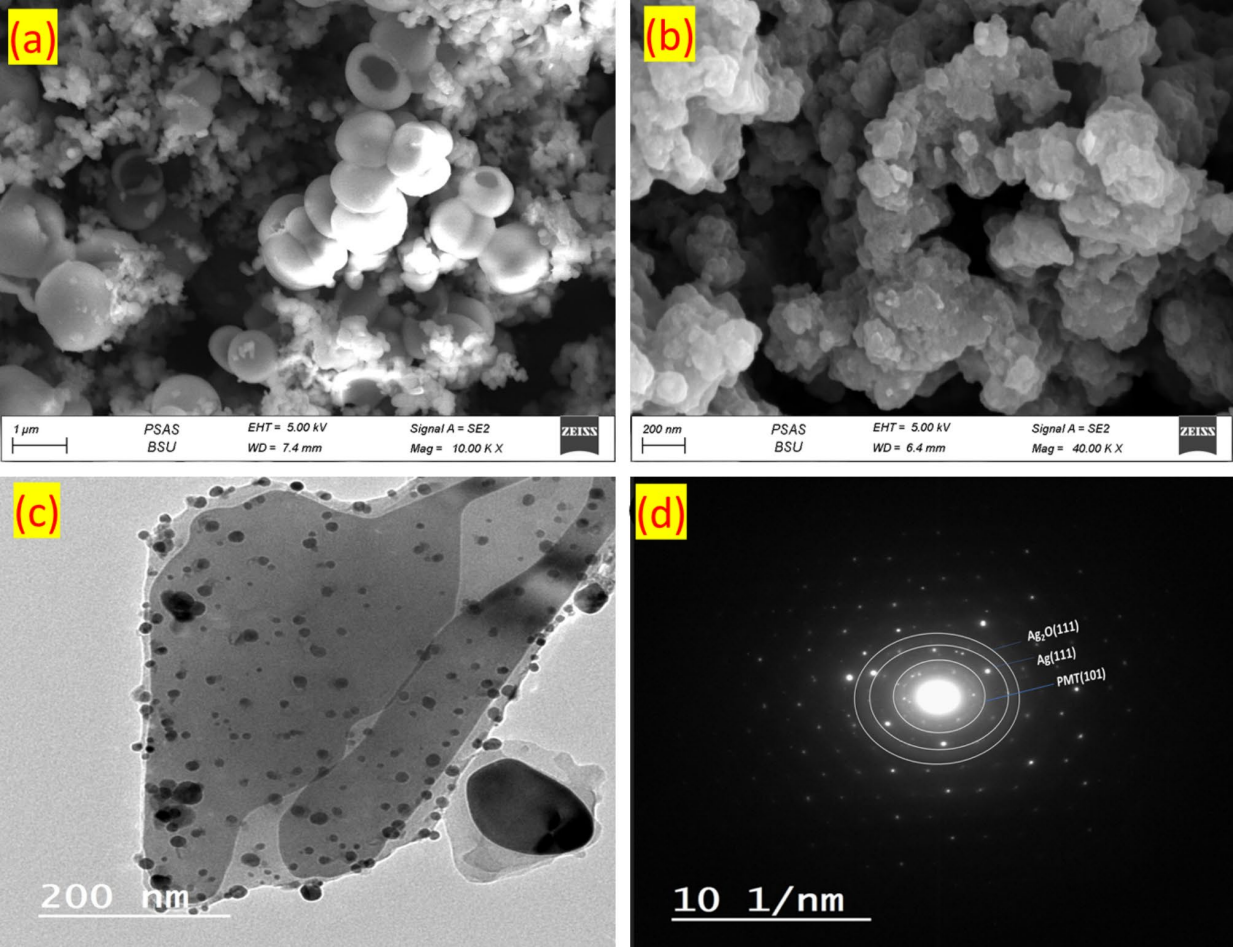
(**a**) SEM image of pristine PMT hollow microspheres, (**b**) SEM image of PMT/Ag-Ag_2_O, (**c**) TEM micrograph of PMT/Ag-Ag_2_O, (**d**) SAED analysis of PMT/Ag@Ag_2_O.

The morphological shapes of the prepared pristine PMT and the PMT/Ag-Ag_2_O nanocomposite were studied using SEM, as displayed in Fig. 3a,b. Only a few hollow spheres and more granular PMT were obtained when the (APS)/(m-toluidine) ratio was 1:1, as shown in Fig. 3a, which is because of the increased consumption of m-toluidine by the APS oxidant^62^. The hole size was measured to be in the range of 0.2–0.53 µm, and the hollow sphere diameter was in the range of 0.95–1.45 µm. The hollow-sphere PMT structures were synthesized by using different ratios of APS to m-toluidine^62,63^. The SEM image of the PMT/Ag–Ag₂O nanocomposite indicates that the nanocomposite exhibits agglomerated, uniformly distributed spherical nanoparticle bundles as seen in Fig. 3b. The particle sizes range from 60 to 220 nm, with a mean size of 170.3 nm, as detailed in Figure S3. TEM analysis was performed to examine the morphology and shape of the prepared PMT/Ag-Ag_2_O, as shown in Fig. 3c. The TEM image reveals distinct spherical nanoparticles on the surface of PMT, with sizes ranging from 10 to 170 nm. The TEM image confirms the successful synthesis of the Ag/Ag_2_O structure on the organic matrix of PMT. These results provide robust evidence for the effective combination of PMT and Ag-Ag_2_O, resulting in the formation of the nanocomposite material. Figure 3d displays the selected area emission diffraction (SAED) of the PMT/Ag-Ag_2_O nanocomposite. The SAED patterns indicate the presence of phase structures in the PMT, Ag, and Ag_2_O nanoparticles. The SAED exhibits diffraction rings, suggesting crystallinity in both silver and silver oxide. The PMT has an amorphous nature with lacks a long-range ordered structure.

**Fig. 4:**
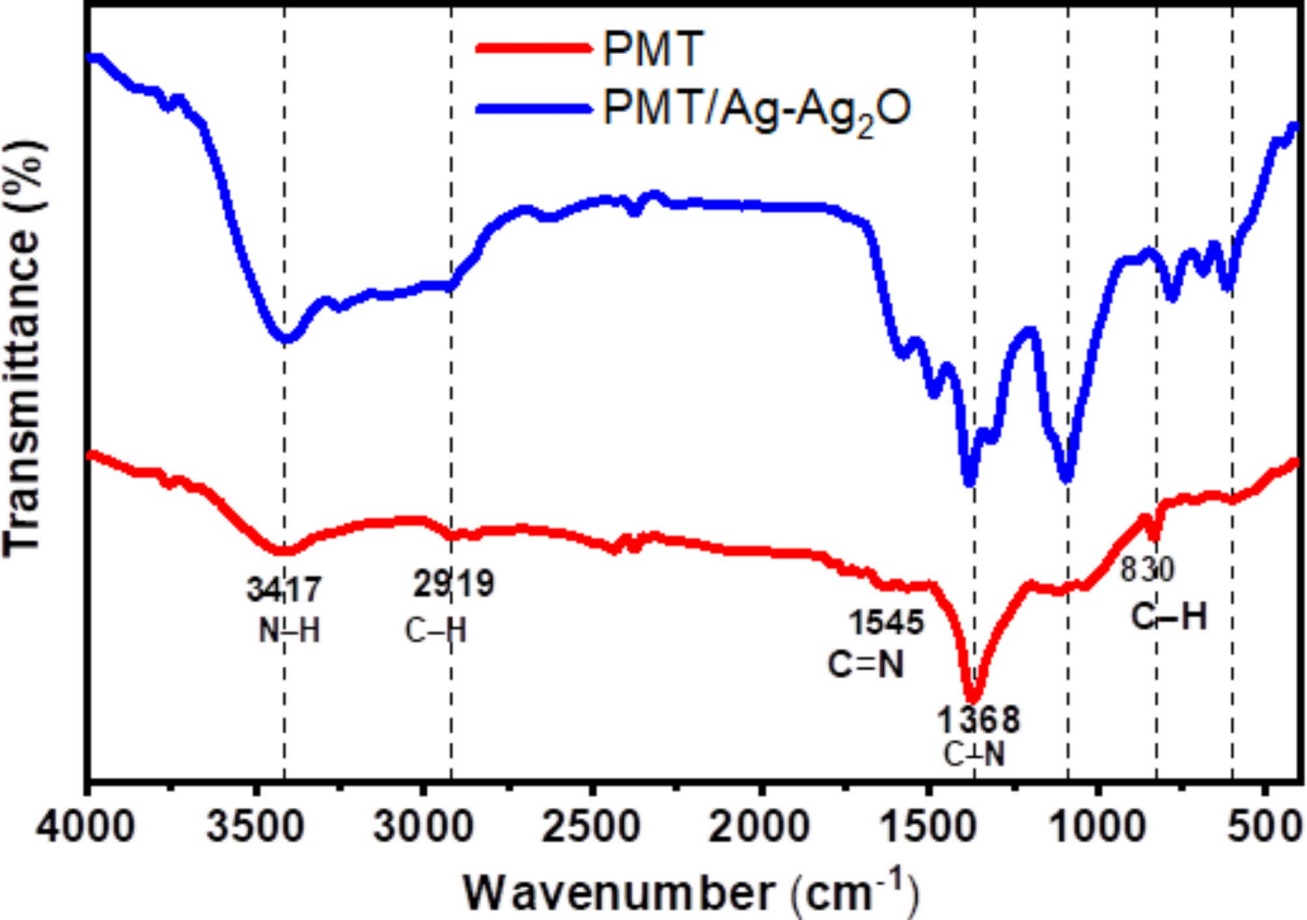
FTIR spectroscopy of the pristine PMT and PMT/Ag-Ag_2_O nanocomposite.

Figure 4 depicts the FTIR characteristic bands of pristine PMT and PMT/Ag-Ag_2_O nanocomposite. As shown, the stretching vibration of the (-NH-) group is assigned to the significant broad band located at 3417 cm^-1^. The band around 2919 cm^-1^ is ascribed to C-H stretching vibrations, while the band pointed at 1112 cm^-1^ is assigned to in-plane C-H bending vibrations in the pure PMT’s quinoid rings^33^. C-N stretching vibrations of benzenoid and quinoid rings located at 1368 cm^-1^ and 1545 cm^-1^, respectively. The spectrum of the PMT/Ag-Ag_2_O nanocomposite also exhibits the characteristic bands of the PMT with a slight shift. The shift supports the interaction occurring between the PMT atoms and Ag/Ag_2_O nanoparticles^33^. Furthermore, the band that appeared at 830 cm^-1^ is attributed to the methyl group attached to the phenyl ring. The stretching mode of Ag–O, which correlates to Ag–O vibration in Ag_2_O, appeared at 605 and 692 cm^-1^. Additionally, the following peak’s presence at 1079 cm^-1^ relates to the vibrations of (Ag-Ag_2_O) PMT caused by metal–oxygen interactions^53^.

### Electrochemical analysis

**Fig. 5:**
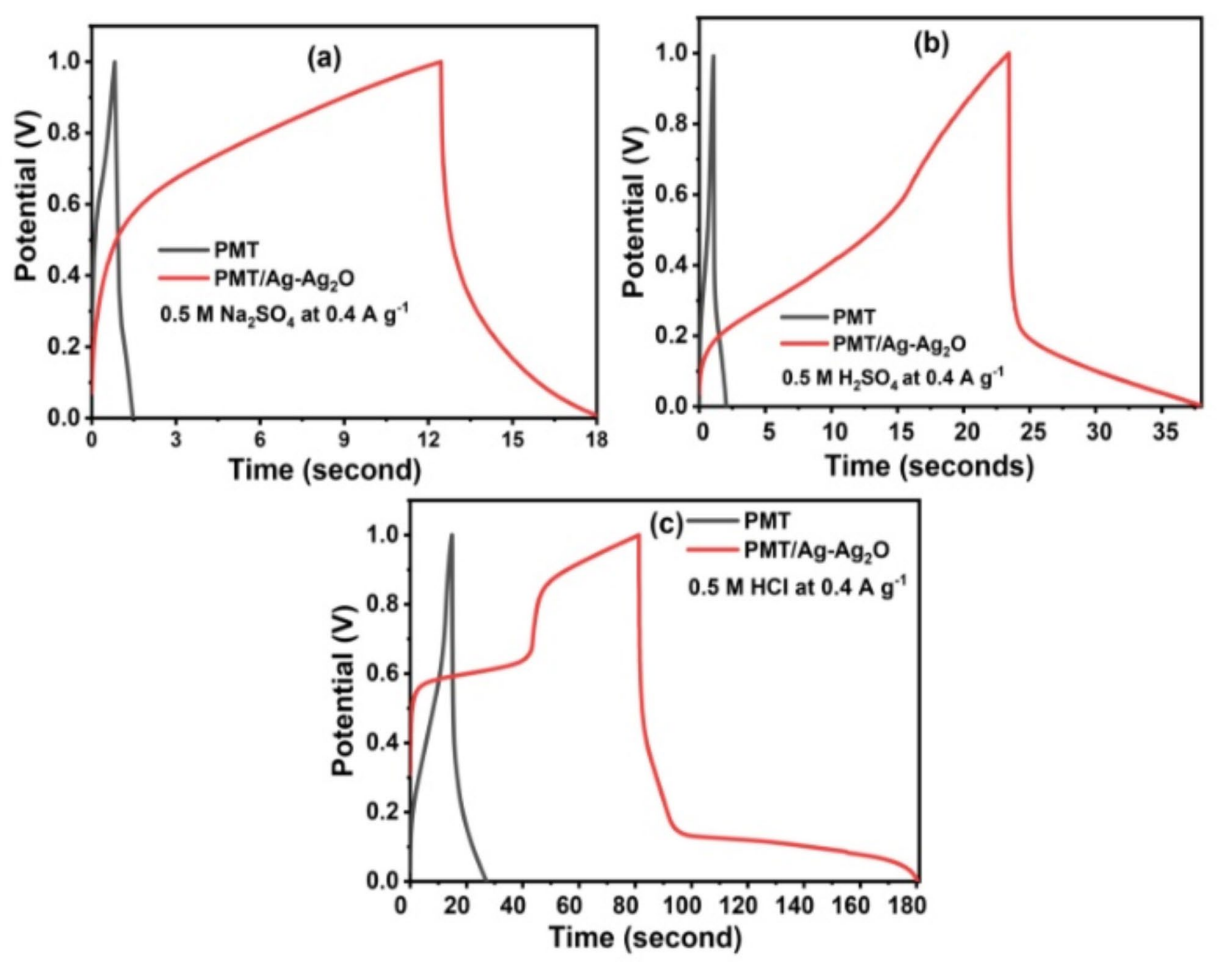
Galvanostatic charge/discharge profiles of pristine PMT and PMT/Ag-Ag_2_O nanocomposite measured at 0.4 A g^-1^ in (**a**) 0.5 M Na_2_SO4, (**b**) 0.5 M H_2_SO_4_, and (**c**) 0.5 M HCl.

The electrochemical performance of the pristine PMT and PMT/Ag-Ag_2_O nanocomposite was evaluated through a GCD test, as depicted in Fig. 5. The GCD test was carried out using 0.5 M Na_2_SO_4_, 0.5 M H_2_SO_4_, and 0.5 M HCl electrolytes at 0.4 A g^-1^. The results indicated the higher capacitive behavior of the PMT/Ag-Ag_2_O nanocomposite compared to the pure PMT in all electrolytes.

The PMT produced a charge/discharge time of about 1/4, 1/15, and 1/6 s that of nanocomposite in 0.5 M Na_2_SO_4_, 0.5 M H_2_SO_4_, and 0.5 M HCl electrolytes at 0.4 A g^-1^, respectively, as depicted in Fig. 5. The nanocomposite exhibited longer charge and discharge times compared to PMT in various electrolytic solutions^64,65^. This can be attributed to the presence of a silver and silver oxide nanoparticles, which have positive effect on charge transfer and increases the number of redox sites available during the charging and discharging processes. Furthermore, the high electrical conductivity of nanocomposite helps to reduce the internal resistance of pseudocapacitive materials. This promotes the diffusion of charges throughout the electrodes, resulting in more efficient charge transfer. The nanocomposite in HCl presented the highest charge/discharge time owing to the highest electric conductivity and smallest hydration spheres of the HCl ions compared to Na_2_SO_4_ and H_2_SO_4_^66,67^. The C_sp_ of the pristine PMT and PMT/Ag-Ag_2_O nanocomposite was calculated using the following equation^33^

4 $${\text{C}}_{\text{sp}}=(4\text{ I }/\text{m}\left(\frac{\Delta \text{E}}{\Delta \text{t}}\right))$$

Here, I present the applied current (A), $\left(\frac{\Delta \text{E}}{\Delta \text{t}}\right)$ presents the slope of the discharge curve, and m (g) is the mass of the electroactive material on each electrode. The voltage window was determined by considering the observed voltage drops in the GCD curves.

The C_sp_ of pristine PMT was calculated to be 3.2, 8.5, and 71 F g^-1^ in 0.5 M Na_2_SO_4_, 0.5 M H_2_SO_4_, and 0.5 M HCl, respectively. The PMT/Ag-Ag_2_O nanocomposite C_sp_ was to be 32,104, and 443 F g^-1^ in 0.5 M Na_2_SO_4_, 0.5 M H_2_SO_4_, and 0.5 M HCl, respectively. All were calculated at a current density of 0.4 A g^-1^. The high capacitance of PMT/Ag-Ag_2_O nanocomposite is related to the Ag-Ag_2_O structure, which provides the channels that facilitate the electrolyte ions transfer. Ag-Ag_2_O architecture increases the faradaic reaction sites and charge transport efficiency. This is achieved through the synergistic effects of using heterogeneous materials for both the Ag and Ag_2_O^68^. In particular, the combination of three materials, namely PMT, silver, and silver oxide, enhances the overall performance of the supercapacitor.

The CP component has high electrical conductivity and specific energy. The silver oxide component, alternatively, exhibits high C_sp_^69^. Moreover, the silver metal component offers greater operating stability compared to both CPs and silver oxide^70^. The result indicates that the PMT/Ag-Ag_2_O nanocomposite is a highly appropriate electrode for supercapacitor devices.

**Fig. 6:**
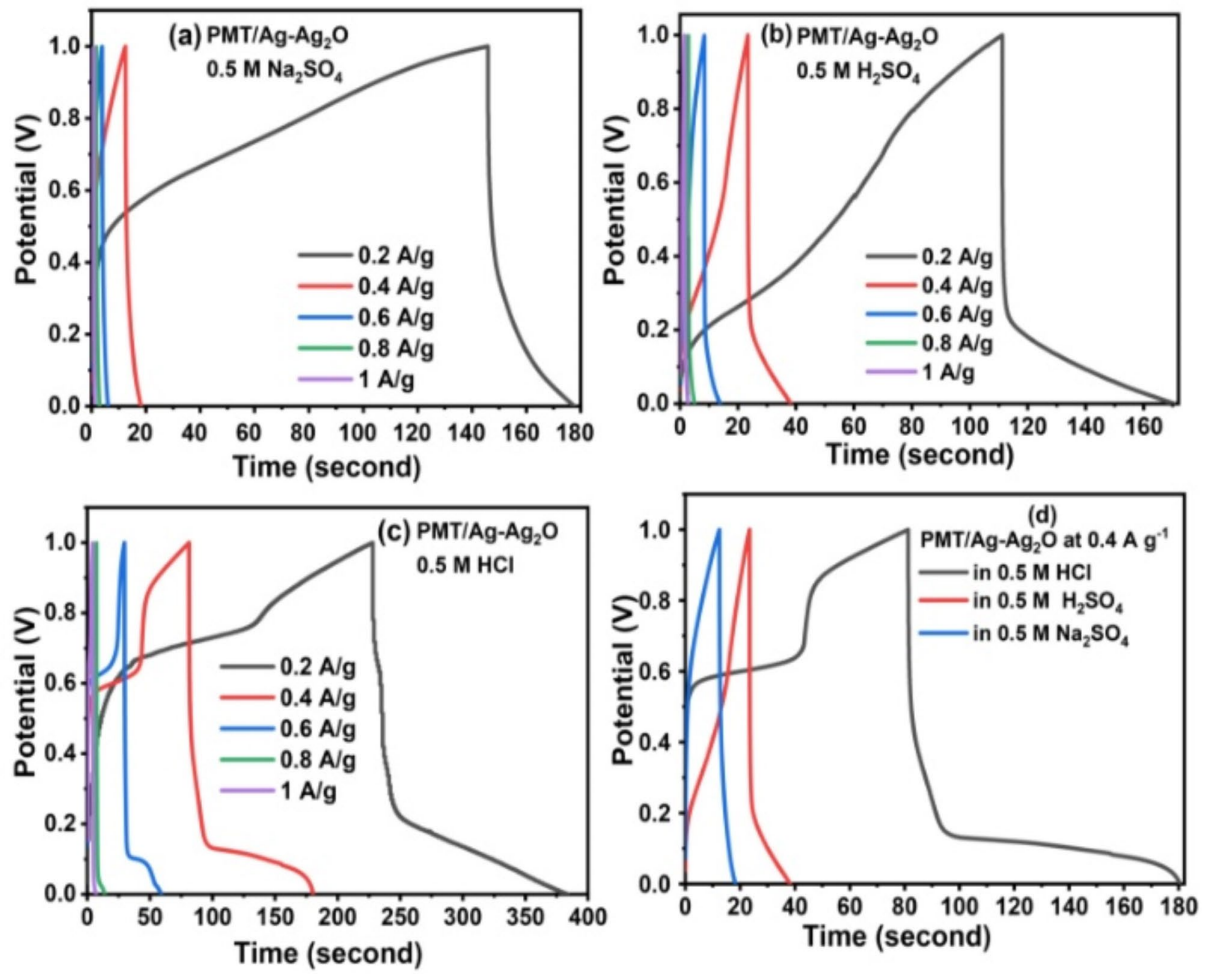
Galvanostatic charge/discharge profiles of PMT/Ag-Ag_2_O nanocomposite measured at different current densities in (**a**) 0.5 M Na_2_SO_4_, (**b**) 0.5 M H_2_SO_4_, and (**c**) 0.5 M HCl. (**d**) GCD curves of PMT/Ag-Ag_2_O nanocomposite in Na_2_SO_4,_ H_2_SO_4_, and HCl at 0.4 A g^-1^.

Figure 6a–c depicts the galvanostatic charge/discharge curves of PMT/Ag-Ag_2_O nanocomposite in different electrolytic solutions at varied current densities of 0.2–1.0 A g^-1^. The analysis of GCD curves indicates that the specific type of electrolyte solution has a considerable impact on how supercapacitors behave in terms of discharge times and capacitance values. Figure 6 shows the lowest charge/discharge time in the Na_2_SO_4_ solution, moderate in H_2_SO_4_, and the highest in HCl. This effect is closely associated with the specific anionic and cationic species present in the electrolyte solution. The results revealed that the C_sp_ was significantly affected by the conductivity, hydrated cationic radius, cationic mobility, and their effects on diffusion and charge/ion exchange^67^. The higher C_sp_ of PMT/Ag-Ag_2_O in HCl electrolyte compared to Na_2_SO_4_ and H_2_SO_4_ electrolytes can be attributed to several factors. Firstly, the smaller hydration sphere of H^+^ and Cl^-^ ions in HCl electrolyte enables higher ion mobility compared to Na^+^ and SO^4-^ ions^66,67^. This increased mobility allows for more efficient ion penetration into the PMT matrix, leading to enhanced capacitance. Moreover, HCl electrolyte exhibits higher ionic conductivity, facilitating better movement of ions within the electrode and improving charge storage and release. On the other side, the Na^+^ ions have a higher hydration sphere radius and lower ionic mobility because of the strong Na^+^-H_2_O interactions generated by the high surface charge density. In comparison to Na^+^, the H^+^ ion possessed a higher ionic conductivity^66,67^. Also, the anionic species’ effect on the electrochemical performance^66^. In comparison to Cl and SO_4_ ions, the Cl ion displayed higher electric conductivity and higher ionic mobility, which explains the significantly superior capacitive behavior. The increased conductivity as well as ionic mobility aid in quick charge transfer, while the reduced hydration sphere radius allows for greater ion adsorption at the interface of the electrolyte/electrode, facilitating the Faradic reaction even more. As a result, the supercapacitor using HCl as an electrolyte has a higher C_sp_ because of the smaller hydrated ions, higher conductivity, and ionic mobility. The GCD curves of PMT/Ag-Ag_2_O nanocomposite at 0.4 A g^-1^ in 0.5 M Na_2_SO_4_, 0.5 M H_2_SO_4_, and 0.5 M HCl are presented in Fig. 6d. The Csp of PMT/Ag-Ag_2_O nanocomposite at around 0.4 A g^-1^ was calculated to be 32 F g^-1^ in 0.5 M Na_2_SO_4_, 104 F g^-1^ in 0.5 H_2_SO_4_, and 443 F g^-1^ in 0.5 M HCl.

The stability of PMT/Ag-Ag_2_O electrode materials in HCl electrolyte is indeed crucial for their practical application. Rahayu et al. have shown that PMT exhibits high conductivity in diluted HCl electrolyte with a concentration of 0.5 M, without destroying the polymer chain structure^71^. Additionally, silver (Ag) is known to demonstrate good stability in the HCl electrolyte due to its position in the reactivity series. This makes the combination of PMT and Ag-Ag_2_O particularly suitable for HCl electrolyte environments. Furthermore, many previous studies have highlighted the effective utilization of appropriate efficient electrode materials such as PPy@ZrO_2_-ZnO and PmT/(Co–Ni) in HCl electrolyte for supercapacitor applications^29,72,73^. Thus, with careful consideration of electrode materials and appropriate design, HCl electrolyte can be effectively used in supercapacitors, leading to impressive capacitance values and retention rates.

The Coulomb efficiency is a critical parameter that indicates the energy efficiency and effective utilization of stored energy in a supercapacitor during charge–discharge cycles. Factors such as irreversible Faradaic reactions, capacitive losses, high self-discharge, and higher internal resistance can contribute to low Coulomb efficiency. To calculate the Coulomb efficiency, the formula ${\upeta}(\text{\%})=\frac{{\text{t}}_{\text{d}}}{{\text{t}}_{\text{c}}}\times 100$ is used, where ${\text{t}}_{\text{c}}$ represents the charging time and ${\text{t}}_{\text{d}}$ represents the discharge time^74–76^. The PMT/Ag-Ag_2_O nanocomposite in HCl electrolyte exhibited the highest Coulomb efficiency of 96.6% at a current density of 0.6 A g^-1^, as depicted in the mentioned Figure S4. The good Coulomb efficiency observed in the PMT/Ag-Ag_2_O nanocomposite can be attributed to the significant reversibility of the redox processes^74,76^. This reversibility indicates highly efficient and reversible charging and discharging reactions, resulting in minimal energy losses and enhanced Coulomb efficiency.

**Fig. 7:**
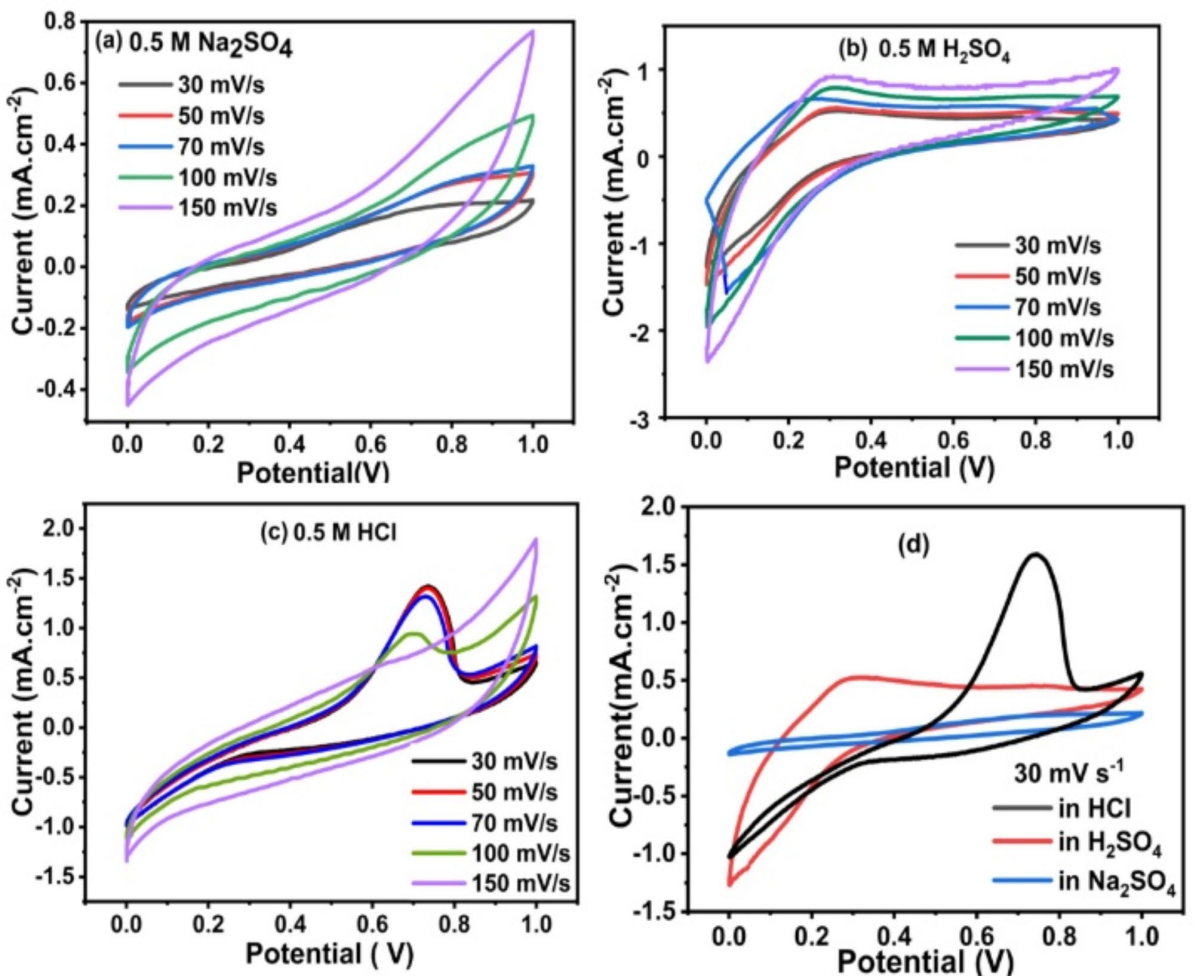
CVs of PMT/Ag-Ag_2_O nanocomposite at different scan rates in (**a**) 0.5 M Na_2_SO_4_, (**b**) 0.5 M H_2_SO_4_, and (**c**) 0.5 M HCl. (**d**) CVs in Na_2_SO_4,_ H_2_SO_4_, and HCl at a scan rate of 30 mV s^-1^.

The CV of PMT/Ag-Ag_2_O nanocomposite at different scan rates in Na_2_SO_4_, H_2_SO_4_, and HCl electrolytes is depicted in Fig. 7a–c. According to the CV curves in Fig. 7, PMT/Ag-Ag_2_O exhibits the representative electrochemical behavior of redox reactions in electrode materials in both 0.5 M Na_2_SO_4_, 0.5 M HSO_4_, and 0.5 M HCl electrolytes. For all electrolytes, it is shown that the oxidation and reduction current increase gradually with an increasing scan rate from 30 to 150 mV s^-1^, demonstrating the desired capacitive behavior of this electrode. Additionally, the CV curves show that PMT/Ag-Ag_2_O has a higher density of stored charges in 0.5 M HCl than that in 0.5 M H_2_SO_4_ and 0.5 M Na_2_SO_4_, as it covers bigger areas and exhibits a more intense redox peak current. Figure 7d displayed the CVs of PMT/Ag-Ag_2_O nanocomposite in Na_2_SO_4,_ H_2_SO_4_, and HCl at a scan rate of 30 mV s^-1^. The area of the CV curve for PMT/Ag-Ag_2_O in 0.5 M HCl at a scan rate of 30 mV s^-1^ is much larger than H_2_SO_4_ and Na_2_SO_4_. This is due to the HCl having the highest mobility and the lowest hydration spheres, which make them enter practically all of the electrode’s pores at the lower scan rates^66,77^. This indicates that it has a higher capacity for electrochemical energy storage. The C_wt_ can be calculated using the next equation

5 $${\text{C}}_{\text{wt}}= \frac{4 {\int }_{{\text{v}}_{1}}^{{\text{v}}_{\text{n}}}\text{i dv}}{\text{ms }\Delta \text{V}}$$

where ${\int }_{{\text{v}}_{1}}^{{\text{v}}_{\text{n}}}\text{i dv}$ is the integrated area under the CV curve, s is the scan rate (V s^−1^) which is applied, $\Delta \text{V}$ is related to the voltage range, and the active material mass on each electrode is denoted m(g)^78,79^. The C_sp_ of PMT/Ag-Ag_2_O was computed from the CV integral area to be 12, 48.8, and 54.8 F g^-1^ in 0.5M Na_2_SO_4_, 0.5 M H_2_SO_4_, and 0.5 M HCl, respectively. The oxidation peak of the PMT/Ag-Ag_2_O electrode displayed in Fig. 7d depicts the reflex reaction of the oxidation of silver to silver oxide at about 0.7 V during the reaction process^80–82^.

6 $$2{\text{ Ag}} + 2{\text{ OH}}^{ - } \leftrightarrow {\text{ Ag}}_{2} {\text{O}} + {\text{ H}}_{2} {\text{O}} + 2{\text{ e}}^{ - }$$

The pseudocapacitance (C_PC_) and electrochemical double-layer capacitance (C_EDL_) together contribute to the total specific capacitance (C_T_) of a material^83,84^. The Trasatti method was used to quantify the contributions of these two capacitance types^83,85^. Understanding the capacitive contributions is crucial for revealing the charge storage mechanism^86^. Trasatti method analyzes the gravimetric capacitance at various scan rates. This method utilizes the following equations^87^

7 $${\text{C}}_{{\text{T}}} = {\text{C}}_{{{\text{PC}}}} + {\text{ C}}_{{{\text{EDL}}}}$$

8 $${\text{1/C}} = {\text{k}}_{{2}} {\text{v}}^{{0.{5}}} + {\text{1/C}}_{{\text{T}}}$$

9 $${\text{C}} = {\text{k}}_{{1}} {\text{v}} ^{{ - 0.{5}}} + {\text{ C}}_{{{\text{EDL}}}}$$

In Eq. 8, the Y-intercept of the linear fit of the inverse of specific capacitance (1/C) plotted against the square root of the scan rate (v ^0.5^) provides the inverse of the total specific capacitance (1/C_T_) for the electrode material. From Eq. 9, the Y-intercept of the linear fit of specific capacitance (C) versus the inverse of the square root of the scan rate (v—^0.5^) gives the electric double-layer capacitance (C_EDL_). Based on Figure S5, the values for C_EDL_ and C_PC_ for the PMT/Ag-Ag_2_O electrode in HCl electrolyte are 2.278 F/g and 163.132 F/g, respectively. This indicates that the PMT/Ag-Ag_2_O electrode exhibits a significant predominance of pseudocapacitance, comprising 98.5% of the total capacitance, while the electric double-layer capacitance accounts for 1.5%.

**Fig. 8:**
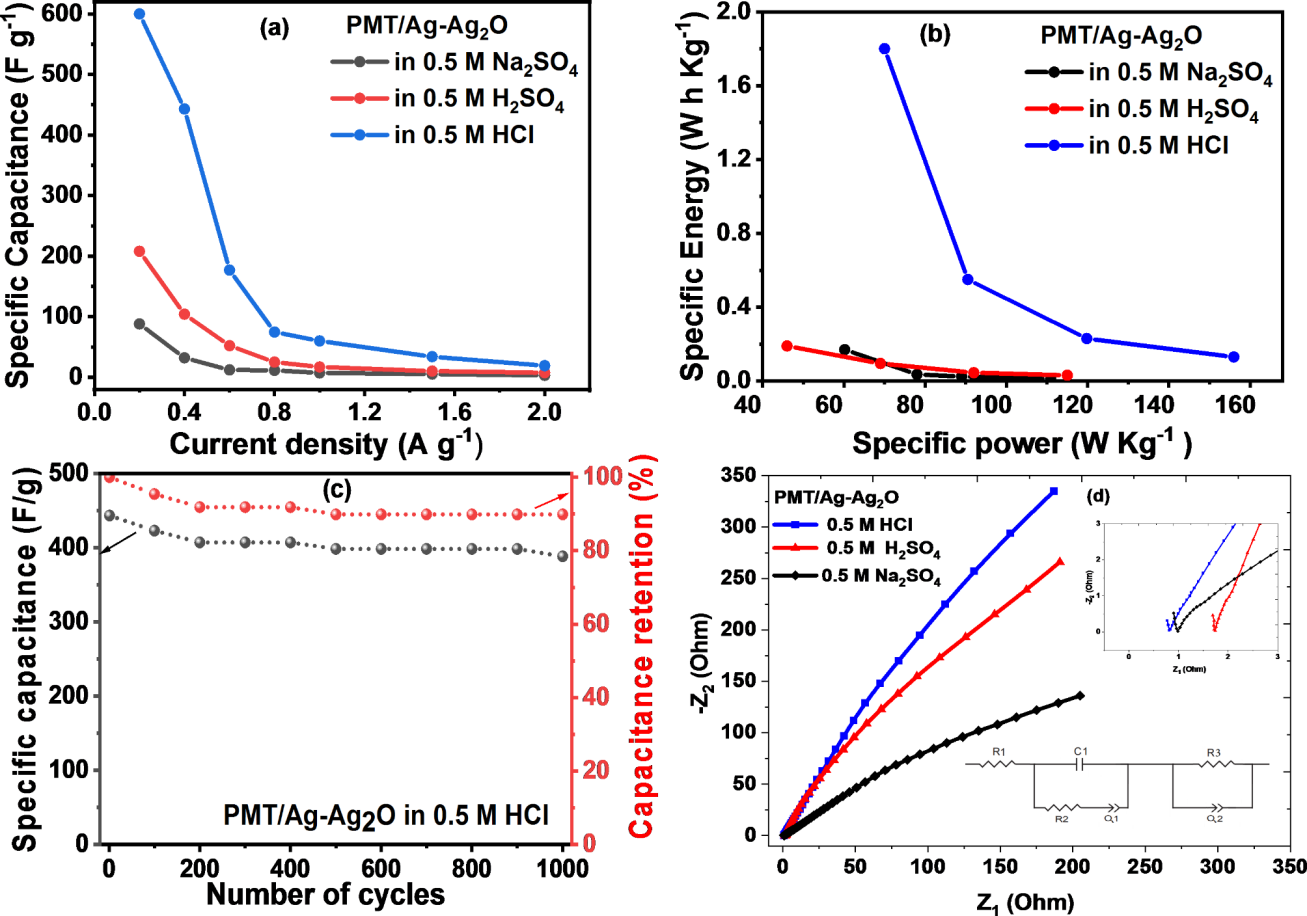
(**a**) C_sp_ against current density, (**b**) Ragon plot profile of PMT/Ag-Ag_2_O nanocomposite in Na_2_SO_4_ and H_2_SO_4_ and HCl electrolytes, (**c**) Cyclic stability and (**d**) Nyquist plot of PMT/Ag-Ag_2_O nanocomposite in Na_2_SO_4_, H_2_SO_4_ and HCl electrolytes (inset is an equivalent circuit).

The relationship between capacitance and current density is shown in Fig. 8a. The capacitance dependency on current density demonstrates that the rate capability of PMT/Ag-Ag_2_O nanocomposite in 0.5 M HCl is superior to that in Na_2_SO_4_ and H_2_SO_4_. In comparison to H_2_SO_4_ (208 F g^-1^) and Na_2_SO_4_ (88 F g^-1^), nanocomposite in HCl has a C_sp_ of 600 F g^-1^ at a current density of 0.2 A g^-1^. The enhanced capacitance of the nanocomposite can be attributed to its unique Ag-Ag_2_O structure, as silver metal in the PMT had high impact as a result of its high ratio in the nanocomposite comparable to the previous study^33^. The the silver nanomaterial in the compsite wit Ag-Ag_2_O ion processh high ratio is a result of the mixed of the APS and AgNO_3_ as mixed oxidants in the polymerization process. The high electrical conductivity of the silver metal ensures efficient charge transfer throughout the charge/discharge process^68^. The increased electrical conductivity of HCl is responsible for its highest capacitive performance. The C_sp_ decreases with an increase in the current density because the active material’s surface at the electrode/electrolyte interface does not have enough time for ion intercalation.

For further study of the electrochemical performance of the PMT/Ag-Ag_2_O electrode, the specific power (P_wt_) and specific energy (E_wt_) plots in the Ragon profile, as shown in Fig. 8b, are given by the following equations^88,89^

10 $${\text{E}}_{\text{wt}} \left(\text{Wh}{\text{ Kg}}^{-1}\right)=\text{A }{\text{C}}_{\text{wt}}{ (\Delta \text{E})}^{2}$$

11 $${\text{P}}_{\text{wt}} \left(\text{W }{\text{Kg}}^{-1}\right)=\text{B }\frac{{\text{E}}_{\text{wt}}}{\Delta \text{t }}$$

Here A and B are constants equal to 0.0347 and 3600, respectively. $\Delta \text{t}$ is the time of discharge and $\Delta \text{E}$ is the potential of discharge after IR drop. The specific energy of a two-electrode supercapacitor can be calculated using the basic formula ${\text{E}}_{\text{wt}}= \frac{1}{2}{\text{C}}_{\text{T}}{ \left(\Delta \text{E}\right)}^{2}$ ^89^. To convert the unit of this formula to watt-hours per kilogram $\left(\text{Wh}{\text{ Kg}}^{-1}\right)$, divide the result by 3600/1000. The resulting formula is ${\text{E}}_{\text{wt}} \left(\text{Wh}{\text{ Kg}}^{-1}\right)= \frac{1}{2\times 3.6} {\text{C}}_{\text{T}}{ \left(\Delta \text{E}\right)}^{2}$ ^90,91^. The C_T_ indicates the supercapacitor cell’s specific capacitance, which equals a fourth of the specific capacitances of the electrode ${\text{C}}_{\text{T}}= {\text{C}}_{\text{wt}} / 4$ ^78^. Taking all these factors into account, The final equation is therefore ${\text{E}}_{\text{wt}} \left(\text{Wh}{\text{ Kg}}^{-1}\right)= \frac{1}{4\times 2\times 3.6} {\text{C}}_{\text{wt}}{ \left(\Delta \text{E}\right)}^{2}$ $=0.0374 {\text{C}}_{\text{wt}}{ \left(\Delta \text{E}\right)}^{2}$ ^92^. To keep this simple, we’ll use 0.0374 as the final constant.

The PMT/Ag-Ag_2_O electrodes’ higher average specific power and specific energy values in Na_2_SO_4_ are 110.9 W Kg^-1^ and 0.17 Wh Kg^-1^, respectively. In H_2_SO_4_, it is 115 W kg^-1^ and 0.19 Wh kg^-1^. For the HCl electrolyte, the higher values of 156 W Kg^-1^ and 1.8 Wh Kg^-1^ are shown.

The retention of C_sp_ concerning charge/discharge cycle numbers up to 1000 cycles at a current density of 0.4 A g^-1^ is studied to assess the cycling stability of the PMT/Ag-Ag_2_O electrode in 0.5 M HCl. The PMT/Ag-Ag_2_O electrode maintained 89.9% of its initial capacitance at 0.4 A g^-1^ after 1000 cycles as seen in Fig. 8c. Throughout the first 400 cycles, there was only a negligible decrease in the retention of the initial capacitance. This can be attributed to various factors, including the activation of redox sites, increased diffusion of PMT/Ag_2_O, and the abundant availability of fresh electrolyte in the sample. The nanocrystalline nature of PMT/Ag-Ag_2_O further facilitated enhanced accessibility of electrolyte ions, promoting efficient diffusion^93^. The consistent kinetics of ion contact within the electrode structure contributed to the overall stability of the nanomaterial during the 500–1000 cycle range. Figure S6 illustrates the GCD behavior of the PMT/Ag-Ag_2_O electrode in 0.5 M HCl for cycle 1, cycle 500, and cycle 1000. This figure demonstrates the electrode’s strong stability, particularly in the range of cycles 500 to 1000. This stability is evident from the consistent discharge profiles and minimal changes in capacitance over the extended cycling period. Previous studies have suggested that a partially irreversible phase change occurring after the redox process could be the primary cause of the observed capacitance degradation within this range. Nevertheless, the PMT/Ag-Ag_2_O electrode showcased excellent long-term cyclic performance overall.

Figure S7 displays the Ragone plot of PMT/Ag-Ag_2_O in Na_2_SO_4_, H_2_SO_4_, and HCl, alongside other storage units for comparison. The electrode based on PMT/Ag-Ag_2_O demonstrates a significant advantage in energy density. Additionally, its targeted power density surpasses that of some alternative battery types. This exceptional electrochemical performance can be attributed largely to the presence of PMT/Ag_2_O. The abundance of active sites and efficient axial electron transport in PMT/Ag-Ag_2_O enhance the kinetics of electrochemical processes and accelerate the penetration of H^+^ ions.

The electrochemical impedance spectroscopy (EIS) of the PMT/Ag-Ag_2_O nanocomposite in 0.5 M Na_2_SO_4_, 0.5 M H_2_SO_4_, and 0.5 M HCl electrolytes is presented in Fig. 8d. A Nyquist plot is used to represent the EIS data. In the plot, the real part of the impedance (Z_1_) is shown on the x-axis, while the imaginary part (Z_2_) is displayed on the y-axis. The dimensions of the axes are equal for orthonormal scales in Nyquist plots. This equality allows for easily visualizing the semicircles and tilted line angles without distortion. As a result, this approach facilitates a more effective and accurate analysis of electrochemical systems and aids in selecting appropriate equivalent electrical circuits.

The Nyquist plot features a small semicircular arc at high frequencies followed by a straight line at lower frequencies. The EIS data were fitted to an equivalent circuit using EC-lab software (version 10.40), as inserted in Fig. 8d. The equivalent circuit includes solution resistance (R_1_), charge transfer resistance (R_2_), surface film resistance (R_3_), double-layer capacitance (C_1_), and constant phase elements (Q_1_, Q_2_). The impedance response of the PMT/Ag-Ag_2_O nanocomposite in the 0.5 M HCl electrolyte demonstrates the lowest resistances observed among the tested electrolytes. Specifically, the calculated R_1_ in 0.5 M HCl is 0.71 Ω, which is lower than the values in the other two electrolytes (1.67 Ω for H_2_SO_4_ and 0.87 Ω for Na_2_SO_4_). This reduction in solution resistance indicates that HCl offers higher conductivity^81^. Moreover, the straight line observed in the low-frequency region is closer to the imaginary part for the 0.5 M HCl solution compared to the other electrolytes, suggesting improved charge transfer kinetics. The charge transfer resistance (R_2_) values are reported as 2.4 Ω for HCl, 3.57 Ω for Na_2_SO_4_, and 5.07 Ω for H_2_SO_4_. This demonstrating that charge transfer is most favorable in HCl, while the kinetics are slower in the other electrolytes. Hence ion transport at the electrode–electrolyte interface is more efficient in HCl, facilitating faster charge separation and transport compared to the other electrolytes. These findings suggest that the PMT/Ag-Ag_2_O electrode exhibits a higher charge storage capacity in the 0.5 M HCl electrolyte^82^.

Finally Table 1 provides a comprehensive comparison of specific capacitance values between this study and many previous works^29,33,94–100^**.**The findings of this study reveal a significantly higher specific capacitance of 443 F g^-1^. In contrast, the PANI/Ag_2_O-Ag paper reported a specific capacitance of 145.2 F g^-1^, while the Ag@Ag_2_O/RGO paper reported a specific capacitance of 119 F g^-1^ ^33,97^.

**Table 1:** A comparison between our results study with previous works.

Nanomaterial	Current density (A g^-1^)	Electrolyte	Specific capacitance (F g^-1^)	References
PMT/(Co–Ni)	0.6	HCl	308	^29^
PANI/Ag_2_O-Ag	0.4	HCl	145.2	^33^
Ag_2_O/PPy	–	KOH	293	^94^
PANI/Ag-rGO	0.5	H_2_SO_4_	379	^95^
Ag_2_S-Ag_2_O-Ag/P2ABT	0.3	HCl	92.5	^96^
Ag@Ag_2_O/RGO	11 mA g^−1^	KOH	119	^97^
PVA/AgAlO_2_@rGO	o.5	Na_2_SO_4_	273.4	^98^
NrGO-Ag@Fe_3_O_4_	0.4	KOH	263.9	^99^
PANI/Ag/cotton fiber electrode	0.5	–	154	^100^
PMT/Ag-Ag_2_O	0.4	HCl	443	This work

## Conclusions

Eventually, the pristine PMT and PMT/Ag-Ag_2_O samples were prepared by the photo-polymerization method, and for further investigation of the nanomaterials, structural, morphological, and optical analyses were carried out. Using three different electrolyte solutions, the electrochemical performance of the nanomaterials was studied using GCD, CV, and EIS techniques. The pristine PMT showed hollow sphere particles, and the PMT/Ag-Ag_2_O nanocomposite showed spherical core–shell structures on the PMT surface. The C_sp_ of pristine PMT and PMT/Ag-Ag_2_O were determined to be 3.2 and 32.1 F g^-1^, respectively, in the Na_2_SO_4_ electrolyte. In H_2_SO_4_, the C_sp_ was measured to be 8.5 F g^-1^ for pristine PMT and 104 F g^-1^ for PMT/Ag-Ag_2_O. Furthermore, in HCl, the C_sp_ was found to be 71.1 F g^-1^ for pristine PMT and 443 F g^-1^ for PMT/Ag-Ag_2_O. For the PMT/Ag-Ag_2_O electrode in HCl, the average specific power and specific energy values are 156 W Kg^-1^ and 1.8 Wh Kg^-1^, respectively. The nanocomposite maintained the capacitance after 1000 cycles to reach 89.9% of its initial value.

## Electronic supplementary material

Below is the link to the electronic supplementary material.

Supplementary Material 1

## Data Availability

The data that support the findings of this study will be made available from the corresponding author upon reasonable request.
