# Supplementary material for: One pot synthesis of poly m-toluidine incorporated silver and silver oxide nanocomposite as a promising electrode for supercapacitor devices
Source: Sci Rep. 2025 Jan 21;15:2698. doi: 10.1038/s41598-024-84848-5 (PMC11750978; doi:10.1038/s41598-024-84848-5)
Supplement: Supplementary file 1 — Supplementary Material 1 [file 41598_2024_84848_MOESM1_ESM.docx]

**One pot synthesis of poly m-toluidine incorporated silver and silver oxide nanocomposite as a promising electrode for supercapacitor devices**

Doaa Essam ^1,2,4*^, Ashour M. Ahmed ^3,4^, Ahmed A. Abdel-Khaliek ^2^, Mohamed Shaban ^5^, Mohamed Rabia ^1^

^1^ Nanomaterials Science Research Laboratory, Chemistry Department, Faculty of Science, Beni-Suef University, Beni-Suef, Egypt.

^2^ Physical Chemistry Laboratory, Chemistry Department, Faculty of Science, Beni-Suef University, Beni-Suef 62514, Egypt.

^3^ Physics Department, College of Science, Imam Mohammad Ibn Saud Islamic University (IMSIU), Riyadh 11623, Saudi Arabia.

^4^ Nanophotonics and Applications Lab, Physics Department, Faculty of Science, Beni-Suef University, Beni-Suef 62514, Egypt.

^5^ Physics Department, Faculty of Science, Islamic University of Madinah, P. O. Box: 170, Al Madinah Al Monawara 42351, Saudi Arabia.

Coreeesponding auther (D.E.): doaae4657@gmail.com


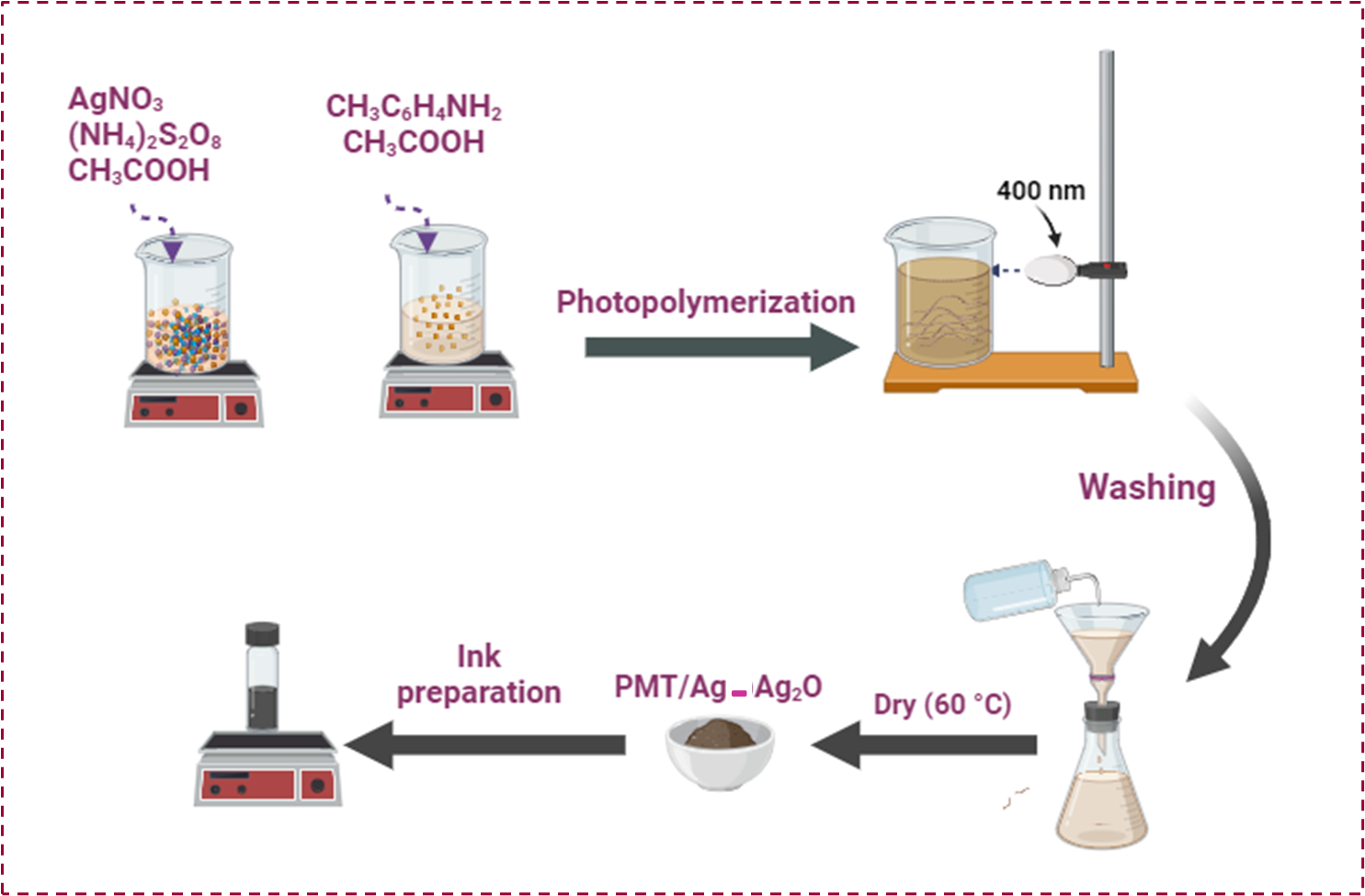


Figure S1. Synthesis of PMT/Ag-Ag_2_O nanocomposite through the photo-polymerization method.


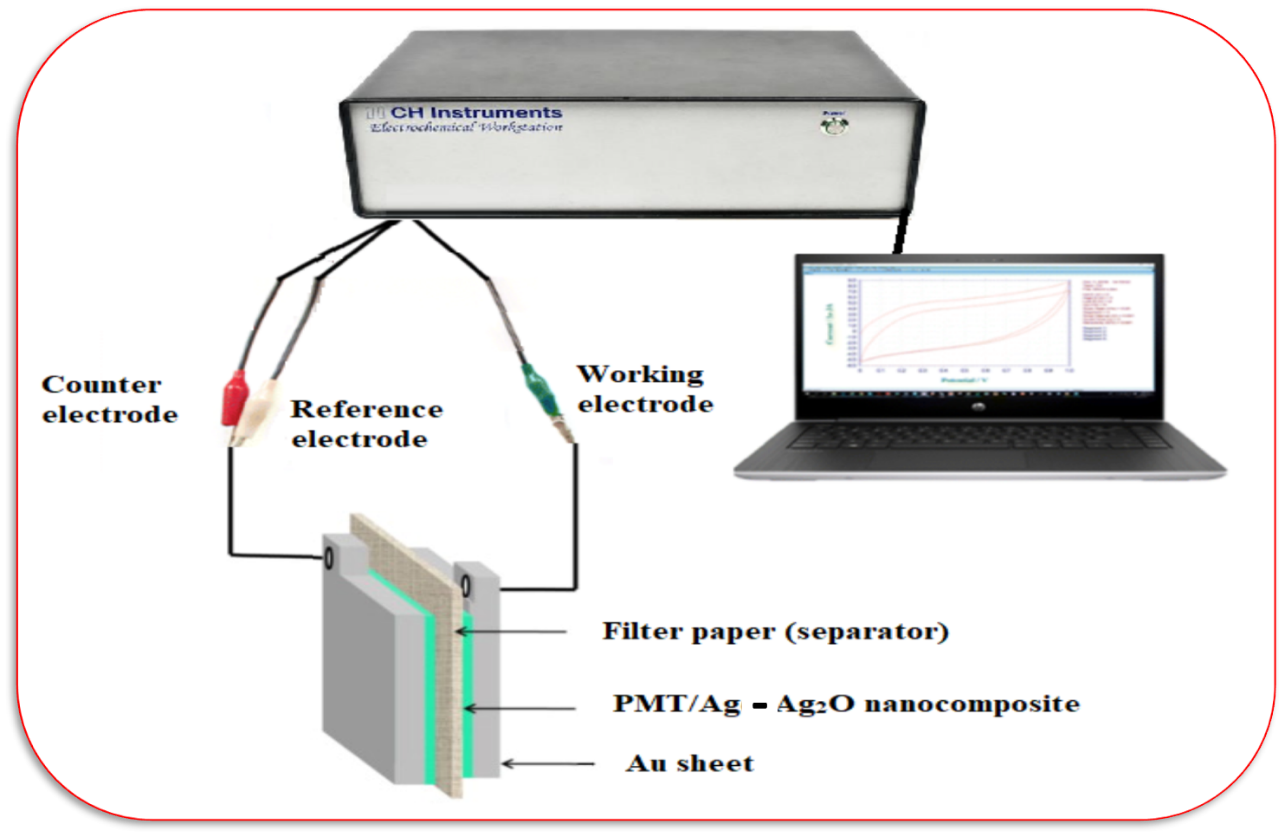
Figure S2. The schematic representation of the electrochemical measurement setup.

Figure S3. (a) SEM image (b) and particle size distribution of PMT/Ag-Ag_2_O nanocomposite.

Figure S4. The Coulombic efficiency of PMT/Ag-Ag_2_O in 0.5 M HCl with different current densities from 0.2 to 2 A g^-1^.


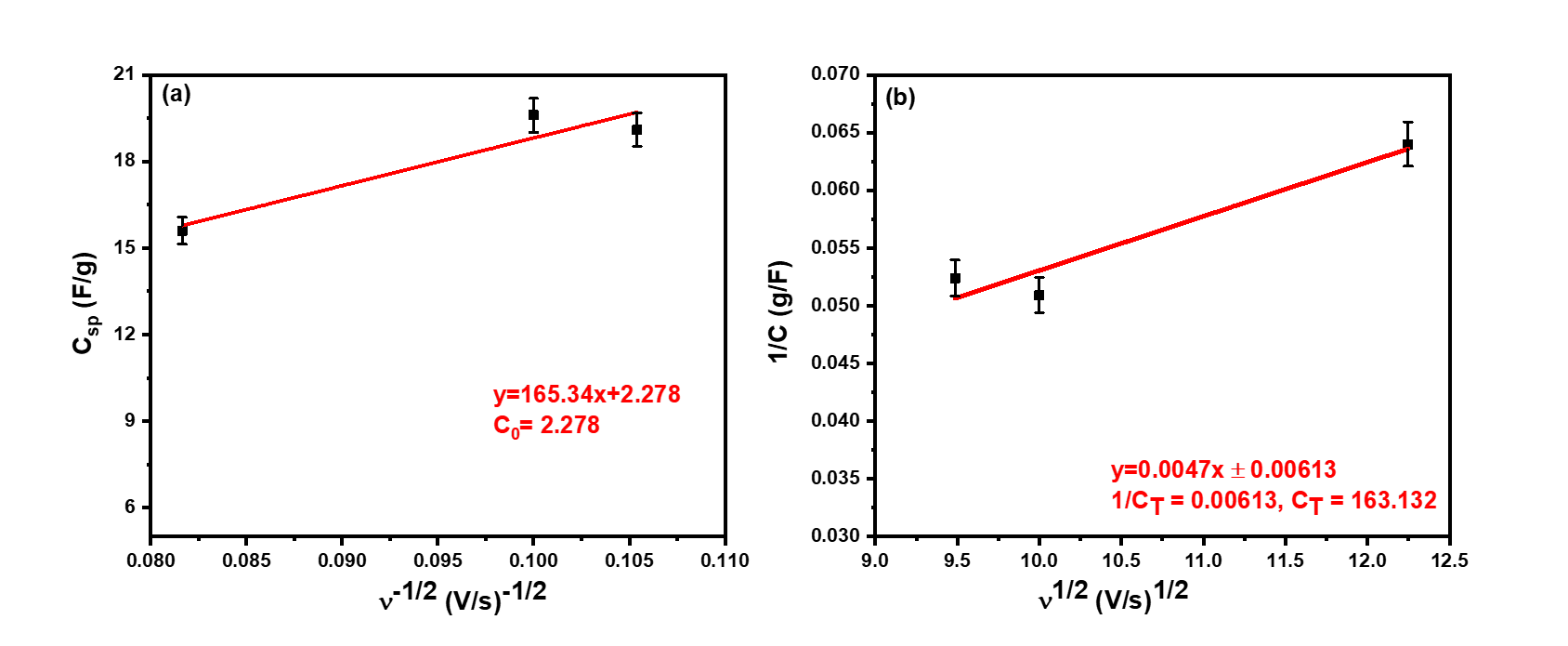


Figure S5. (a, b) Trasatti’s method analysis: (a) Gravimetric capacitance (C) vs. inverse of the square root of the scan rate (v ^-0.5^). (b) Reciprocal of gravimetric capacitance (C^-1^) vs. square root of the scan rate (v^0.5^).

Figure S6. The GCD of the PMT/Ag-Ag_2_O electrode in 0.5 M HCl for cycle 1, cycle 500, and cycle 1000.


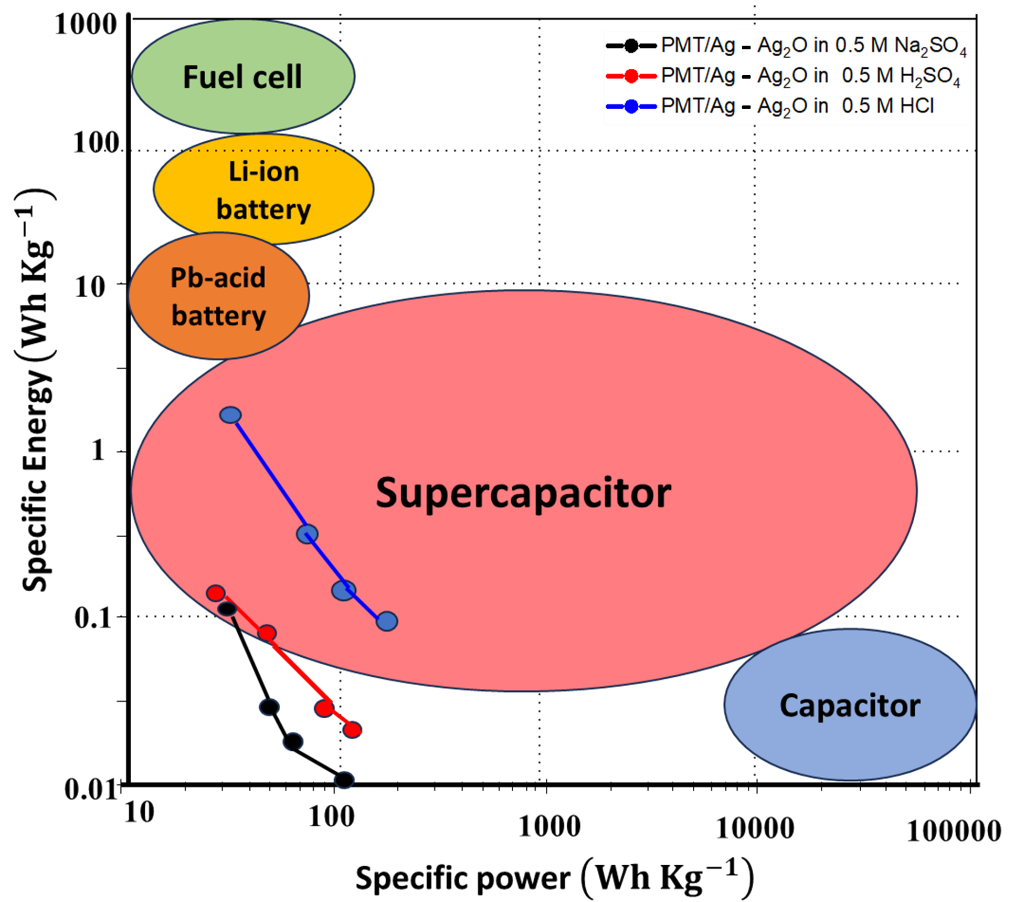


Figure S7. Ragone plot of PMT/Ag-Ag_2_O in Na_2_SO_4,_ H_2_SO_4_, and HCl.
